# A multilevel investigation of leader–member exchange differentiation’s consequences: A moral disengagement perspective

**DOI:** 10.3389/fpsyg.2022.969346

**Published:** 2022-09-15

**Authors:** Amer Ali Al-Atwi, Elham Alshaibani, Ali Bakir, Haneen M. Shoaib, Mohanad Dahlan

**Affiliations:** ^1^Department of Business Administration, Al-Muthana University, Samawah, Iraq; ^2^Department of Business Administration, University of Karbala, Karbala, Iraq; ^3^University of Business and Technology, Jedda, Saudi Arabia

**Keywords:** LMX differentiation, customer-oriented deviance, moral disengagement, LMX differentiation bases, banking sector

## Abstract

We examine the effects of leader–member exchange (LMX) differentiation on team members’ outcomes (customer-oriented constructive and destructive deviant behaviors) by using team moral disengagement as a psychological mechanism mediating this relationship and LMX differentiation bases (i.e., performance and personal liking) moderating the relationship. Analysis of multilevel data collected from 289 frontline employees organized into 76 finance-related customer service teams shows that LMX differentiation significantly reduced team moral disengagement only when the performance basis was high, and that the negative relationship between LMX differentiation and team moral disengagement was significant only when the personal liking basis was low. Furthermore, we found that the LMX bases moderated the indirect effect of LMX differentiation on team members’ outcomes through team moral disengagement. The findings advance team moral disengagement as a novel mechanism for cross-level relationship between LMX differentiation and team members’ outcomes at the individual level, and project differentiation bases as a condition under which LMX differentiation unpacks the reasons for team members’ favorable or unfavorable responses. They reveal LMX differentiation as a complex and multifaceted phenomenon, whose essence can only be understood if examined from multiple levels. We also contribute to the literature by revealing the cognitive pathway through which LMX differentiation may be associated with team members outcomes.

## Introduction

The increasing prevalence of organizations’ adoption of work teams has led leadership researchers to intensify their efforts in understanding the leadership processes that emerge at the team level (Burke et al., 2011). One of these more team-specific extension processes by which the team leader forms different patterns of exchange relationships (ranging in quality from low to high) with team members, is called leader member-exchange (LMX) differentiation (Henderson et al., 2009; Van Knippenberg, 2017). LMX researchers belatedly realized that the focus of early work was supposed to give weight to the consequences of team leaders developing different quality relationships with members in their teams (i.e., LMX differentiation; Liden et al., 2006) and not just focus on the dyadic relationship between a team leader and team member (LMX); as LMX differentiation was the origin of the emergence of LMX theory (Henderson et al., 2009). Consequently, we witness much LMX research predominantly focused on LMX differentiation in the relevant literature today. This is a promising development that opens new possibilities for LMX research and re-ignites interest in the consequences of its influence at various theoretical levels (e.g., Bolino and Turnley, 2009; Henderson et al., 2009; Ma and Qu, 2010; Epitropaki and Martin, 2015; Martin et al., 2018; Matta and Van Dyne, 2020).

Compared to the volume of studies that dealt with the outcomes of LMX differentiation for the entire team, little efforts was devoted to studying its outcomes for the individual members (Erdogan and Bauer, 2010; Anand et al., 2011). Investigating how differential treatment affects members’ outcomes has been fragmented (Kauppila, 2016). Some studies have indicated that LMX differentiation negatively affects team members’ outcomes (e.g., Hooper and Martin, 2008; Henderson et al., 2009; Dong et al., 2020). For example, Hooper and Martin (2008) found that LMX differentiation reduces team members’ feelings of well-being and commitment. Other studies have found that LMX differentiation does not necessarily bring bad things to members, but rather it may achieve good things (e.g., Erdogan and Bauer, 2010; Le Blanc and González-Romá, 2012). For example, Erdogan and Bauer (2010) found that differentiation has the potential to motivate members to help one another. However, a number of studies have not found any significant relationship between LMX differentiation and team members’ work outcomes (e.g., Liden et al., 2006; Liao et al., 2010; Gooty and Yammarino, 2016). To date, the picture emerging from these studies regarding the relationship between LMX differentiation and members’ outcomes is unclear and quite inconsistent (Kauppila, 2016). Further research addressing the potential mechanisms and contingencies of these relationships may thus be necessary.

Light needs to be shed on the restricted view of theoretical perspectives used to explain LMX differentiation’s outcomes to help solve the puzzle of these inconsistent results (Harris et al., 2014). According to Martin et al. (2018), researchers have relied heavily on specific theoretical lenses such as the theories of justice, social comparison, and social identity to explain the effects of LMX differentiation. However, despite the usefulness of this research, the search for alternative theoretical lenses is likely to provide new insights into understanding LMX differentiation’s outcomes. We develop and test a theoretical model of LMX differentiation effects on team members’ outcomes by using moral disengagement theory (Bandura, 1986) in conjunction with legitimacy and justice lens in social hierarchy theory (Tyler, 1997, 2006; Halevy et al., 2011). Integrating these two perspectives allows us to unravel the cognitive mechanism (moral disengagement) and conditions (differentiation bases) under which LMX differentiation translates into members’ outcomes.

Moral disengagement theory deals with cognitive mechanisms that allow individuals to detach from their internal moral standards and act unethically without distress (Detert et al., 2008). We believe that this perspective can help explain the consequences of LMX differentiation by showing how moral disengagement can act as a cognitive mechanism to transform differential behavior by the leader into unacceptable behaviors of members in the workplace. A significant part of such members’ behaviors is manifested in customer-oriented constructive and destructive deviant behaviors, which will be elaborated further in the “Theory and hypothesis development” section.

**Figure 1:**
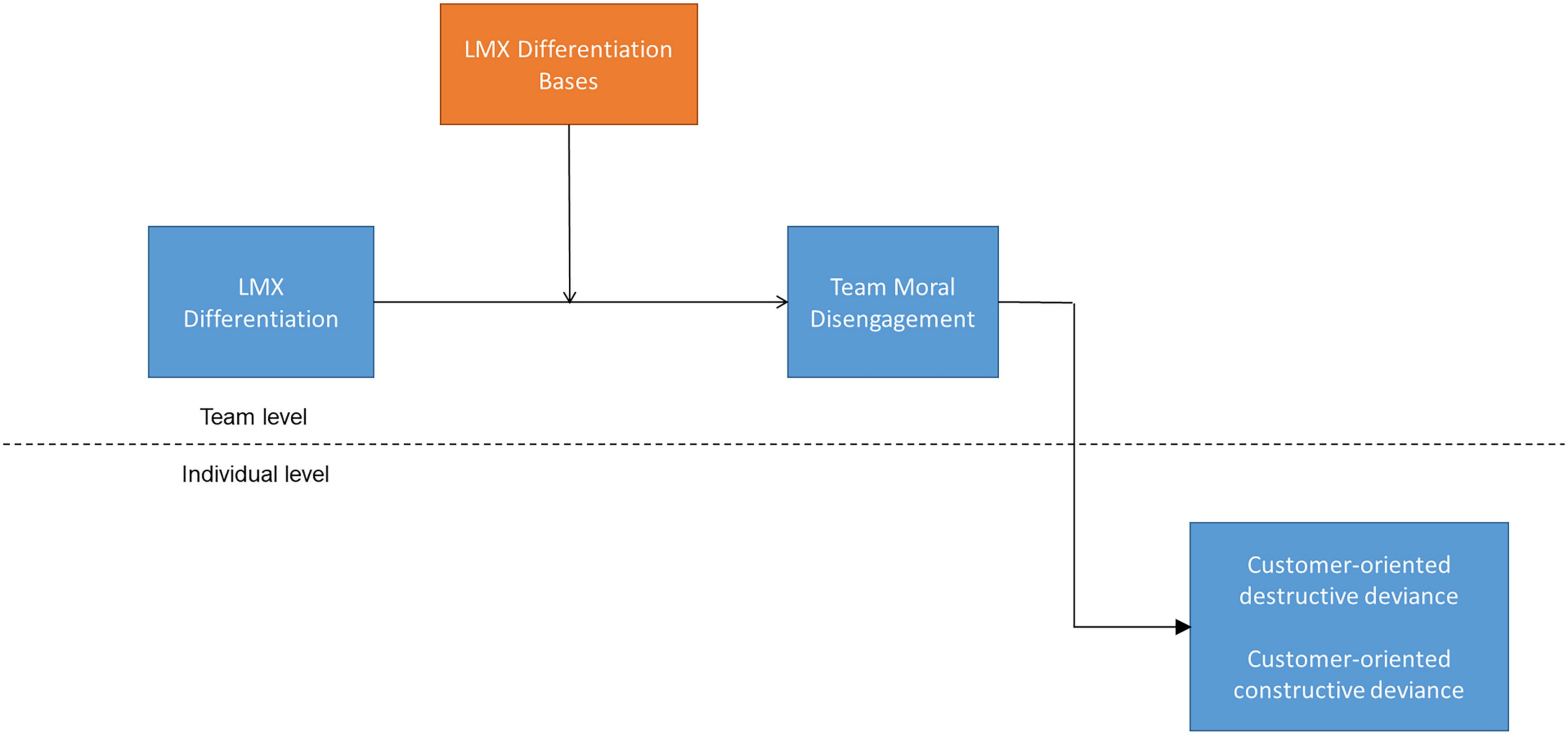
Hypothesized model.

The social hierarchy theory, on the other hand, suggests that some members will respond favorably and others unfavorably to hierarchical differentiation based on the leader’s legitimacy of the differentiation base used (Halevy et al., 2011). Studies identified two bases of LMX differentiation: performance and personal liking (e.g., Dulebohn et al., 2012; Han et al., 2021). Performance base refers to task-related factors such as members’ skills and competencies while personal liking base expresses non-task-related personal factors such as kinship, likeability, and similarity (Chen et al., 2015; Han et al., 2021). We expect that the performance basis is viewed positively as legitimate and the personal liking basis is viewed negatively as biased (Tyler, 1997). In summary, building on moral disengagement theory and social hierarchy theory, we propose that LMX differentiation interacts with performance bias and personal liking bias to affect members’ deviant customer-oriented behaviors through the indirect effect of team moral disengagement (Figure 1).

By making this proposition, we potentially contribute to the literature on LMX differentiation in two ways. First, we examine LMX differentiation from a multilevel perspective. Most LMX differentiation research has been confined to the group level, with an evident dearth in the use of multilevel frameworks (Yammarino et al., 2005; Anand et al., 2011; Harris et al., 2014). Our paper supports growing research addressing the multilevel nature of LMX differentiation that helps create more comprehensive predictions about outcomes at the individual level (Harris et al., 2014). Second, we consider alternative perspectives that offer new theoretical insights into the effects of LMX differentiation (Robin et al., 2017) through integrating moral disengagement theory (Bandura, 1986) with social hierarchy theory (Halevy et al., 2011). Using both perspectives guide us to (1) advance team moral disengagement as a novel mechanism for the cross-level relationship between LMX differentiation and members’ outcomes, contributing to our understanding of the mechanisms that lead to differential effects (Matta and Van Dyne, 2020); and (2) state that the indirect relationship between LMX differentiation and members’ outcomes through team moral disengagement is conditional on the two differentiation bases: performance and personal liking. Thus, projecting differentiation bases as a condition under which LMX differentiation unpack the reasons as to why some members respond favorably and others unfavorably.

In addition, we also contribute to the literature on moral disengagement in two ways. First, research examining leadership factors that prompt members to disengage morally is relatively under-explored compared with other factors (Johnson and Buckley, 2015). For example, Ashforth and Anand (2003) point out that unethical behavior and associated moral disengagement mechanisms may become normalized because of a permissive ethical climate nurtured by leadership and organizational structures and processes (see also, Moore, 2008; Huang and Yan, 2014; Martin et al., 2018). However, in contrast to the burgeoning research on moral disengagement at the individual-level of analysis, only a dearth of research has examined work-team moral disengagement (e.g., Huang and Yan, 2014). Furthermore, to our knowledge, no study addresses the role of LMX differentiation. By addressing LMX differentiation, we are answering two calls: (1) to adopt multilevel approaches to study the antecedents of moral disengagement and (2) to study leadership behavior that may lead to higher levels of moral disengagement among members rather than focusing only on leadership behavior that reduces it, such as ethical leadership (Newman et al., 2020).

Second, our contribution is not limited to the antecedents of moral disengagement but also includes the consequences. Most of the research studies the effect of moral disengagement of employees on the results that affect their colleagues, the departments in which they work, or the leader with whom they communicate, all of which reflect the internal consequences in the workplace. According to Johnson and Buckley (2015), there is a significant gap in the moral disengagement literature in external interpersonal exchanges such as employees communicating directly with customers. Our study addresses this gap by testing the relationship between moral disengagement and deviant customer-oriented behaviors.

In addition to filling a theoretical gap in the literature on LMX differentiation and its outcomes, we hope that this study will come up with findings that would support the literature in encouraging leaders to realize that differential treatment of team members might not necessarily be bad if it is aimed at making efficient use of limited resources and time, and at achieving a better fit between employees’ abilities and their work requirements (Anand et al., 2011; Martin et al., 2018). We further hope that the findings would further alert leaders to develop quality relationship with team members, as past studies show that members are susceptible to differentiation and will question the basis on which the leader favors some members and not others. Team members respond favorably to differentiation, which they perceive, based on performance-related factors, and unfavorably when based on personal liking. Organizations may then reap the benefits of differentiation and avoid incurring the costs of employee moral disengagement and loss of customers.

### Theory and hypothesis development

We couple moral disengagement theory (Bandura, 1986) and social hierarchy theory (Halevy et al., 2011) to propose a cross-level model to understand the mechanism through which LMX differentiation affects individual outcomes. Moral disengagement is conceptualized by Bandura (1986, 1999) as a set of cognitive mechanisms that allow individuals to disassociate from their internal moral graenstandards and behave unethically without feeling guilt or distress. Johnson and Buckley (2015) argued that moral disengagement is an inherently interpersonal phenomenon, and because of high levels of interpersonal proximity within a group, a morally disengaged group member is likely to cause other members to disengage morally due to social contagion. In addition, Ashforth and Anand (2003) argued that unethical behavior and the moral disengagement mechanisms associated with it become normalized in organizations owing to a permissive ethical climate facilitated through leadership, organizational structures, and processes (see also, Moore, 2008; Huang and Yan, 2014; Martin et al., 2014).

According to social hierarchy theory, hierarchy in organizations has multiple asymmetric dimensions along which individuals can be differentiated, such as power and control over resources, status, and participation and contribution to group tasks and activities (Halevy et al., 2011). Han et al. (2021) indicated that LMX differentiation could be one of the potential sources in creating a social hierarchy among team members. Closeness to the leader, support, access to resources, and trust due to the high quality of the relationship with the leader can be considered by individuals as a sign of high status. In contrast, a poor relationship with the leader is considered a sign of low status (Dulebohn et al., 2012). Halevy et al. (2011) presented a lengthy exposition on how hierarchy enhances team and organizational performance. They argued that, among other things, hierarchy creates a psychologically rewarding environment better than any other social arrangement by establishing a clear chain of command. The effect of hierarchical differentiation is not always beneficial; rather, the relationship can be positive or negative and typically depends on the influence of specific moderators (Halevy et al., 2011; Han et al., 2021). Halevy et al. (2011) further argued that the hierarchal social arrangement is more likely to have positive effects on the group and organizational processes when it has high rather than low legitimacy (see also Kelman, 2006). Consistent with the view of power as a social affordance (Keltner et al., 2008), Tyler (2006) argued that legitimacy entails that followers are likely to obey authoritative demands because they personally feel they ought to do so. Team members’ internalized commitment to legitimate hierarchies increases organizational performance in several complementary ways, such as: sustaining higher levels of cooperation at little or no costs, decreasing friction and conflict, positively effecting performance through complementarity and coordination, and also influencing the individual-level processes that are affected by hierarchy (Gneezy and Rustichini, 2000; Tyler, 2006; Halevy et al., 2011).

### LMX differentiation and moral disengagement: Two bases of LMX differentiation

Our study suggests that it is likely that members will morally disengage in teams in which the leader develops differential LMX relationships with their members based on illegitimate factors and is unlikely to disengage morally if the LMX relationship is based on legitimate factors. The various factors that determine the formation of LMX differential relationships between leaders and their members within a team are called the bases of LMX differentiation (Chen et al., 2015). Extant research has identified two bases of LMX differentiation that are relevant to the development of high-quality LMX: performance and personal liking (e.g., Dulebohn et al., 2012; Han et al., 2021). Performance basis refers to task-related factors on which the leader relies to develop high-quality relationships with members, such as members’ skill, competencies, and level of performance (Chen et al., 2015). The personal liking basis expresses social and personal factors unrelated to the task the leader depends on in developing high-quality relationships with members, such as personal relationships, liking, and similarity (Han et al., 2021).

We think that leaders can create perceived difference in attitude through their behavior in how they treat team members. The more consistent and clear these behaviors are in their signs, the more accurate the team members’ awareness of them will be. Team members may mentally ask what are the reasons behind the fact that a member is close to and valued by the leader and not others (Martin et al., 2015). With the passage of days, members will discover that the reasons for this differentiation are related to performance factors or personal factors. For example, when team members notice that one of the relatives or friends of the leader within the team did not receive a specific reward or privilege due to his/her poor performance or lack of competence, this will be a clear message that performance-related factors are the basis in differentiating dealings and vice versa. Therefore, when leaders differentiate LMX relationships based on performance-related factors, they are sending a message to team members that the member who contributes the most to the team’s effectiveness deserves more positive attention and recognition based on his/her good work performance (Matta and Van Dyne, 2020). This makes team members feel that the differentiation in treatment is legitimate because it is based on justified and proper foundations (Halevy et al., 2011; Han et al., 2021). Conversely, when leaders differentiate LMX relationships based on issues that have no direct relationship with performance, team members will perceive this treatment as inequitable and unjustified (Van Breukelen et al., 2012; Matta and Van Dyne, 2020). Studies have shown that members view differential treatment as fair only when it is justified based on the level of effort and efficiency, and they will feel uncomfortable if the leader’s differential treatment is not based on these grounds (e.g., Sias and Jablin, 1995; Sias, 1996). It is further suggested that LMX differentiation based more on job-irrelevant attributes than actual contributions through task performance or organization citizen behavior (OCB) violates the rule of justice and damages the leader’s position with members (Scandura, 1999; Chen et al., 2015).

The moral disengagement literature indicates that a sense of fairness in the workplace can reduce employees’ unethical behavior by not leading them to disengage morally and vice versa (e.g., Liu and Berry, 2013; Hystad et al., 2014; Loi et al., 2015). For example, Hystad et al.'s (2014) study indicated that the feeling of unfairness led employees to practice deviant behaviors, as the perceptions of injustice led to employees feeling that the formal rules and procedures were illegitimate and thus invalidated. In their current meta-analysis, Ogunfowora et al. (2021) found that when employees perceive fairness in the interpersonal treatment of leaders and in the outcomes and procedures of decisions in the organization, they are not likely to disengage with their moral standards and engage in deviant behavior at work.

In addition, Chen et al. (2015) indicated that leaders who treat their members differently by relying more on job-irrelevant attributes would incite high sensitivity among members to social comparison information, which will lead to a work context in which negative feelings such as anger and envy prevail. Past studies have found that individuals who experience negative feelings tend to disengage morally from their inner principles (Fida et al., 2015; Ye et al., 2021). On the other hand, differentiation based on tasks or personal matters may be an important aspect in shaping team members’ impressions and expectations toward the moral considerations of their team leaders (Van Breukelen et al., 2012). When a leader develops differential LMX relationships with members based on performance and ability, members are likely to develop feelings of integrity and trust toward the leader (Chen et al., 2015) and a sense that the leader acts fairly (Matta and Van Dyne, 2020). These positive characteristics are among the essential things that members rely on to judge the moral behavior of their leaders (Bonner and McLaughlin, 2014). In addition, studies have confirmed that moral leaders make members more aware of moral concerns and more reluctant to entertain morally disengaged thinking (Huang and Yan, 2014; Moore et al., 2019; Ogunfowora et al., 2021).

Han et al. (2021) drew on LMX differentiation, LMX differentiation bases, and social hierarchy theory (Halevy et al., 2011) in testing the effect of the interaction of LMX differentiation with LMX bases on group social undermining. They found that LMX differentiation was negatively related to group social undermining when it was based on the group members’ performance and positively related when based on the leader’s personal liking. Strongman (2014) also argued that moral disengagement is central to the manifestation of social undermining in personal and professional group behaviors, particularly dehumanization (see also, Bandura, 1999). Furthermore, Ogunfowora et al. (2021) found that employees who experience abusive supervision and organizational politics are significantly likely to employ moral disengagement. Drawing on social exchange and social cognitive theories, Valle et al. (2018) argued that employees experiencing negative LMX behavior, such as abuse which may include non-verbal abuse (e.g., dislike), are more likely to morally disengage (Bandura, 1986, 1999) to justify their voluntary counter-productive workplace deviant behaviors. As such, employees’ behaviors undermine the organization’s norms and threaten the well-being of its members. Valle et al. (2018) called for the use of the social cognitive construct of moral disengagement to help explain the cognitive processes that may underlie the verbal and non-verbal (e.g., dislike) abusive LMX organizational deviance relationship.

These and other studies provide sufficient evidence to propose the following two hypotheses regarding the relationship between LMX differentiation, LMX differentiation bases, and team members’ moral disengagement.

*Hypothesis 1*: Performance basis moderates the relationship between LMX differentiation and team moral disengagement, such that the relationship is less positive when performance basis is high compared to low.

*Hypothesis 2*: Personal liking basis moderates the relationship between LMX differentiation and team moral disengagement, such that the relationship is more positive when personal liking basis is high compared to low.

### Moral disengagement and team members’ deviant customer-oriented behaviors

Moral disengagement stems from the idea of how an individual can engage in immoral behavior without feeling distressed (Bandura et al., 1996). Previous research has demonstrated that moral disengagement can lead to a variety of negative behaviors that may not be consistent with one’s own internal moral standards, such as unethical decision-making (Detert et al., 2008; Moore et al., 2012), CWB (Astrove et al., 2015; Fida et al., 2015), unethical behaviors (Barsky, 2011; Keem et al., 2018), deviant behaviors (Hystad et al., 2014; Huang et al., 2017), and undermining behaviors (Duffy et al., 2012).

Deviant behaviors resulting from moral disengagement are not limited to the internal interactions that the individual conducts with colleagues and superiors within the organization but may extend to include external interactions such as deviant behaviors toward customers (Johnson and Buckley, 2015). Organizations today urgently need to adopt ways to prevent frontline employees from engaging in unethical behavior toward customers, as such behavior may entail many costs for the organizations (Zaal et al., 2019). According to Yan et al. (2021), these organizations deter their frontline employees from morally disengaging from their internal values and principles and always feel self-punishing, not only through the threat of severe organizational punishment but also through their increased desire to achieve organizational goals.

Previous literature dealt with two types of deviant behaviors practiced by frontline service employees toward customers (e.g., Morrison, 2006; Hunter and Penney, 2014; Dahling and Gutworth, 2017). The first type is destructive deviation, also called customer-directed counter-productive workplace behavior. Customer-oriented destructive deviance refers to voluntary actions by employees that harm or aim to harm the organization’s customers and include, for example, employee rudeness, unresponsiveness, and mistreatment of customers (Spector and Fox, 2005; Hur et al., 2018). The second type is customer-oriented constructive deviance, which refers to any instance where a frontline service employee intentionally violates a formal organizational policy, regulation, or prohibition with the primary intention of achieving a customer benefit (Gong et al., 2020). According to Gong et al. (2020), although constructive behaviors benefit customers and, as a result, may benefit the organization, in various circumstances, these behaviors can be destructive for the organization. For example, employees may engage in behaviors intended to benefit the customer; however, their actions may end up being unfair to other customers.

Although past studies did not test the effect of moral disengagement on deviant behavior toward customers, these studies have greatly enriched the role of moral disengagement in motivating individuals to practice deviant behavior toward their colleagues or the organization (Astrove et al., 2015; Huang et al., 2017). When moral disengagement between team members is activated for any reason, this will give the members the green light to violate the organization’s norms and policy without hesitation and without feeling any psychological pain when the opportunity arises. When morally disengaged members are on the front lines of service and have daily personal exchanges with customers, they are likely to practice deviant behavior toward internal individuals such as their colleagues and leaders and include external individuals such as customers (Johnson and Buckley, 2015). Role requirements for service jobs expose employees’ work context and pressures that may increase the likelihood of engaging in deviant behavior toward customers (Hunter and Perreault, 2006). Team members cannot always direct their retaliation to the source of frustration because the source may be in a higher or more powerful position or unavailable in some circumstances. Instead, members may direct their retaliation toward those who are less powerful, like customers, especially when their work is in direct contact with them (Bordia et al., 2010). Moral disengagement allows these individuals to believe that ethical standards do not apply in the current situation. They fail to realize the ethical implications of their behavior and therefore make unethical decisions toward customers without realizing any pressure or guilt (Yan et al., 2021). It is not just that morally disengaged employees sometimes defy workplace rules in the form of deviant behavior that hurts customers in retaliation against the organization or its leaders. Instead, we believe that morally disengaged employees sometimes defy organizational rules in the interest of a party such as customers because they are motivated enough to oppose practices they see as stagnant, ineffective, or even dangerous to those around them (Dahling and Gutworth, 2017). For example, morally disengaged frontline service employees may try to justify their violation of workplace rules for the benefit of the customer with various considerations, such as objecting to the organization’s policy and its unfairness toward customers or achieving the interests of the organization in the long run (Priesemuth, 2013; Gong et al., 2021). According to the above logical discussion, we propose the following:

*Hypothesis 3*: Moral disengagement will positively affect customer-oriented destructive deviance (CODD).

*Hypothesis 4*: Moral disengagement will positively affect customer-oriented constructive deviance (COCD).

### Moderated mediation effects

Our model assumes that the interaction between LMX differentiation and its bases (i.e., performance and personal liking) relates to individuals’ customer-oriented deviant behaviors through team moral disengagement. A large number of studies dealt with moral disengagement as a mediating variable that explains the relationship between leadership predictors and work behaviors (e.g., Palmer, 2013; Moore, 2015). For example, Palmer (2013) found that moral disengagement mediated the relationship between leader behavior and follower ethical behavior at the individual-level of analysis. Moreover, Hodge et al. (2013) found that moral disengagement mediated the relationship between controlling coaching styles and higher levels of anti-social behavior toward team members and opponents. Moore et al. (2012) have similarly found that moral disengagement mediates the relationship between how ethical an employee’s leader is and the likelihood they will engage in unethical workplace behavior. Valle et al. (2018) called upon social exchange and social cognitive theories to understand the mechanism through which normative work behavior is displaced by deviant behavior. They focused on moral disengagement as a mediating variable between abusive supervision and deviance and on LMX differentiation as a contextual moderating variable that they proposed may affect the nature of this mediated relationship. The results from their empirical study reported a statistically significant positive relationship between abusive supervision and moral disengagement and moral disengagement and organizational deviance, which suggests that moral disengagement mediated the relationship between abusive supervision and organizational deviance. Huang and Yan (2014) reported that ethical leadership led to lower levels of group moral disengagement and that group moral disengagement mediated the relationship between ethical leadership and the collective organizational deviance of team members.

When leaders treat their members differently based more on job-irrelevant attributes, they will damage their personal attractiveness to members, violate the rules of justice, and create personal conflict among colleagues (Chen et al., 2015). Cues of unwarranted preferences and favoritism resulting from the leader’s differential treatment based on performance-irrelevant issues (Matta and Van Dyne, 2020) can provide an easy justification for members trying to disengage their principles morally. Therefore, when the leader’s behaviors negatively activate people, moral disengagement can lead them to perceive that engaging in deviant practices may be an appropriate strategy for dealing with that behavior (He et al., 2017). On the contrary, a performance basis of LMX differentiation makes team members feel that the leader’s behavior is desirable, appropriate, and, therefore, legitimate and fair (Han et al., 2021). Previous studies have shown that the experience of legitimacy and fair procedures when dealing with leaders makes people think ethically (Tyler et al., 2008) and encourages them in general to adopt different forms of cooperation, including making additional efforts to help the organization be effective as well as avoiding all actions that could violate its rules and instructions (Tyler and Blader, 2000; Chen et al., 2015).

When personal aspects are vital for LMX differentiation, we expect individuals to behave destructively toward the organization and its customers. Because the leader’s treatment in this way makes team members feel that the leader’s behavior is inappropriate and unsatisfactory, and therefore it is illegitimate behavior (Matta and Van Dyne, 2020; Han et al., 2021). This treatment can make team members feel like victims of the leader’s undesirable behavior, which increases the moral violation, reduces the ability to self-regulate, and ultimately ignites moral disengagement (He et al., 2017). Also, awareness of the illegitimacy and unfairness of authority motivates individuals to think immorally and increases their tendency to resist (Tost, 2011) and break the rules rather than follow them (Tyler et al., 2008). In contrast, the sense of legitimacy of authority arising from the performance basis of LMX differentiation can appeal to team members’ values and moral norms as a means of achieving high levels of positive, voluntary effort that benefits rather than harms the organization (Tyler, 1997; Halevy et al., 2011). According to Tyler et al. (2007), the behavior of authority that creates a sense of legitimacy makes organizational values and norms become part of individuals’ internal value systems and directs their behavior independently of the effect of incentives and punishments, replacing external control with self-control. As a result, these people become self-regulatory, assume the responsibilities associated with those norms and values as aspects of their own motivations, and move away from behaviors that break them in the interest of the organization and its goals. Thus, such individuals are not likely to break its rules and norms to harm its customers or benefit them, hence the following hypothesis.

*Hypothesis 5a*: Performance basis moderates the indirect effect of LMX differentiation on team member’s CODD through team moral disengagement and this indirect effect will be less positive when performance basis is high compared to low.

*Hypothesis 5b*: Performance basis moderates the indirect effect of LMX differentiation on team member’s COCD through team moral disengagement and this indirect effect will be less positive when performance basis is high compared to low.

*Hypothesis 6a*: Personal liking basis moderates the indirect effect of LMX differentiation on team member’s CODD through team moral disengagement and this indirect effect will be more positive when personal liking basis is high compared to low.

*Hypothesis 6b*: Personal liking basis moderates the indirect effect of LMX differentiation on team member’s COCD through team moral disengagement and this indirect effect will be more positive when personal liking basis is high compared to low.

## Materials and methods

### Sample and procedures

In order to investigate the proposed research questions, we collected multilevel data from three large banks. The data collection process targeted service teams working in these banks’ branches located in different provinces. Our study focused on service teams whose members are in regular contact with customers and have working experience practicing frontline duties. These teams work in functional customer service areas, such as banking consulting, checks, currency exchange, bills, current accounts, credit facilities, commercial transfers, documentary credits, and deposits.

We collected our data on-site during participants’ working hours using questionnaires. We sent these questionnaires to 350 frontline employees organized in 86 service teams (86 supervisors of these employees) selected in targeted banks. We excluded six teams because they had less than three participated members and four teams did not return the response of their members. Three of the 292 returned questionnaires were removed due to substantial missing data, thus, yielding a final sample of 289 effective questionnaires from 76 service teams (response rate: 82.5%). Frontline employees were asked to rate their relationship quality with supervisors, team moral disengagement, and deviant customer-oriented behaviors. Among frontline employees, 64.4% were male; 84.4% had attained a diploma’s degree or higher; and the average age of the participants was 37.57 (SD = 9.25). Among supervisors, 75% were male and the average age was 43.11 years old (SD = 5.75); the majority (78.9%) had a bachelor’s degree or above. The service teams ranged in size from 3 to 6 members.

### Measurements

#### Leader–member exchange quality

LMX quality was assessed using the seven-item scale (LMX-7) developed by Graen and Uhl-bien (1995). An example of the items includes “I have enough confidence in my leader that I would defend and justify his/her decision if he/she were not present to do so?” Items were scored on a seven-point Likert scale (1 = not at all; 7 = to a large extent). The Cronbach’s alpha for this measure was.89 in this study.

#### Leader–member exchange differentiation

In line with prior studies (e.g., Chen et al., 2015; Han et al., 2021), we used the standard deviation of the group members’ LMX ratings to calculate LMX differentiation. Our choice to operationalize LMX differentiation was based on Buengeler et al.’s (2021) framework, which indicates that the LMX differentiation measure should be consistent with the theoretical considerations assumed in the study, such as the meaning of LMX in the studied sample. Specifically, our study describes LMX similarly to a key tenet LMX theories (Graen and Uhl-bien, 1995) that a team leader forms different patterns of exchange relationships with team members. These patterns separate team members into two relatively equal groups, one group with high quality, and the other with low LMX quality relationships. This meaning is consistent with the idea of LMX separation in Buengeler et al.'s (2021) framework, which depends on the existence of a form in which the difference is symmetric within the group. Standard deviation is one way to measure LMX as separation (Buengeler et al., 2021).

#### Leader–member exchange differentiation bases

Following prior studies (e.g., Dulebohn et al., 2012; Han et al., 2021), we used performance and personal liking as two main bases of LMX differentiation. We assessed these two bases using items recently developed by Han et al. (2021). For measuring performance based on LMX differentiation, we asked the participants to determine to what extent the group leader treats their team members differently based on three indicators: “the job performance of the team members, members’ contribution to the service team, and members’ value to the service team.” The member responses (Cronbach alpha = 0.94) were aggregated to compute the team-level construct. We assessed personal liking-based LMX differentiation by asking the participants to determine to what extent the team leader treats their team members differently based on three indicators: “team leader’s personal liking, leader’s personal tie, and leader’s personal favor.” We also aggregated the individual responses (Cronbach alpha = 0.96) to team-level. Items of both scales were scored on a seven-point Likert scale (1 = “strongly disagree” to 7 = “strongly agree”).

#### Moral disengagement

We measured moral disengagement using eight items developed by Moore et al. (2012). Following the recommendations of previous studies (Kish-Gephart et al., 2010; Ogunfowora et al., 2021; Yan et al., 2021), we adapted these items to suit the specific situational features. In addition, we benefited from Yan et al. (2021) study, which adapted some of these items to fit the frontline employees’ context. For example, a sample item reads, “Considering the ways organizations in banking industry grossly misrepresent themselves, it’s hardly a sin to inflate your organization’s profile a bit.” As recommended by Newman et al. (2020), we conceptualized team-level disengagement as averaging of members’ level of disengagement. This requires aggregating individual-level ratings of moral disengagement to the team level after ensuring that the aggregation statistics are satisfactory. Response options ranged from strongly disagree (1) to strongly agree (7); the Cronbach’s alpha for this measure was.87.

#### Customer-oriented constructive deviance (COCD)

Customer-oriented constructive deviance was measured using a three-item scale (Dong et al., 2020). Sample items are “I break organizational rules to provide better service to the bank customers.” Response choices on all items are ranged on a 7-point scale from 1 (strongly disagree) to 7 (strongly agree), and Cronbach’s alpha was.91.

#### Customer-oriented destructive deviance (CODD)

We used 7-items developed by Hunter and Penney (2014) to assess customer-oriented destructive deviance. An example item reads, “Made a customer wait longer than necessary.” Each item was scored on a five-point Likert scale [“never” (1) to “every day” (5)]. Cronbach alpha for this scale was 0.88.

#### Control variables

At the team member level, we followed past studies (e.g., Skarlicki et al., 2016) by controlling for key demographic characteristics, including age, gender, and education, which can potentially affect our results. More so, due the consequences of LMX differentiation at the individual-level are likely to be contingent on each individual’s LMX status within the group, it is necessary to take into account individual LMX status in our model. This can be done with the addition of relative LMX (RLMX) as a control at the individual level. By following past studies (e.g., Henderson et al., 2008; Tse et al., 2012), we calculated LMX scores by subtracting the mean individual-level LMX score in a team from the focal member’s LMX score. We followed previous research at the leader and team level by controlling for the leaders’ age, gender, and education (e.g., Dong et al., 2020). We also included group mean LMX (e.g., Sui et al., 2015; Dong et al., 2020) and team size (Alnuaimi et al., 2010; Han et al., 2021) as control variables.

## Results

### Data aggregation

Because our model was targeted at the team level of analysis, we conducted inter-rater agreement (*r*_wg_), and intra-class correlation (ICCs) tests for justifying the aggregation of individual team members’ survey responses to the team level (James et al., 1984; Bliese, 2000). Data aggregation included three variables: performance-based LMX differentiation, liking–based LMX differentiation, and team moral disengagement. The *r*_wg_ scores for these variables were 0.71, 0.71, and 0.75, respectively (above the criteria of 0.70), indicating an acceptable level of agreement among team members (James et al., 1984). Calculation of ICC(1) indicated that the variance between groups was 0.23, 0.42, and 0.33, respectively, which were above the cutoff values (LeBreton and Senter, 2008). In addition, the F ratios associated with the ICC1 value of all our three variables were statistically significant. Calculation of ICC(2) indicated that group perceptions’ reliability was 0.53, 0.74, and 0.65, respectively, which were above the cutoff values (LeBreton and Senter, 2008). Overall, our findings justified aggregating all individuals’ responses to the team level.

### Confirmatory factor analysis (CFA)

We performed a confirmatory factor analysis (CFA) to assess the discriminant validity of our hypothesized model that included six variables: LMX, moral disengagement, performance basis, personal liking basis, COCD, and CODD by using AMOS V.24. Fit indices for the six-factor model (*χ*^2^/(419) = 2.19; CFI = 0.91; RMSEA = 0.06; SRMR = 0.05) were better than other alternative models. Detailed results of the alternative model tests are available on request from the first author.

### Descriptive statistics

Table 1 presents descriptive statistics for the variables and Pearson correlations among them. We noted that the inter-correlations of the key variables were in the expected direction.

**Table 1:** Means, standard deviations, and intercorrelations among study variables.

Variable	*M*	SD	1	2	3	4	5	6	7	8	9
*Individual level*											
1. Gender	0.64	0.47	1								
2. Age	37.57	9.25	0.03	1							
3. Education	4.56	1.09	0.00	−0.10	1						
4. RLMX	0.00	0.56	−0.08	−0.15*	0.04	1					
5. Performance basis	5.12	1.45	0.03	0.13*	−0.07	−0.22**	1				
6. Personal liking basis	3.62	1.74	−0.13*	−0.05	0.06	0.04	−0.14*	1			
7. Moral disengagement	3.10	1.23	−0.01	−0.04	−0.09	0.05	−0.13*	0.32**	1		
8. COCD	2.60	1.41	−0.14*	−0.06	−0.07	0.06	−0.13*	0.23**	0.44**	1	
9. CODD	1.40	0.60	0.01	−0.01	−0.06	0.02	−0.06	0.16**	0.42**	0.43**	1
*Team level*											
1. Team size	3.80	1.06	1								
2. Leader gender	0.75	0.44	−0.22	1							
3. Leader age	43.11	5.76	−0.15	−0.02	1						
4. Leader education	5.28	1.08	0.01	−0.08	−0.07	1					
5. LMX mean	3.76	0.52	−0.22	0.08	0.22	−0.07	1				
6. LMX differentiation	0.13	0.11	0.37**	−0.27*	−0.06	−0.07	−0.39**	1			
7. Performance basis	5.14	1.02	−0.06	0.16	0.08	0.12	0.43**	−0.27*	1		
8. Personal liking basis	3.63	1.37	−0.02	−0.09	−0.10	0.05	−0.52**	0.20	−0.22	1	
9. Team moral disengagement	3.09	0.89	0.07	−0.10	−0.12	−0.02	−0.41**	0.17	−0.30**	0.36**	1

* *p* < 0.05;

** *p* < 0.01.

Individual level *N* = 289, team level *N* = 76. RLMX, relative leader-member exchange; LMX, leader–member exchange; COCD, customer-oriented constructive deviance; OCDD, customer-oriented destructive deviance. For gender, 1 = male, 0 = female.

### Tests of hypotheses

Given that the data used in this study is nested (members nested within teams), we ran multilevel analyses using MPlus 7 (Muthén and Muthén, 2012). In order to assess the variance in the dependent variables (COCD and CODD) due to clustering, we first estimated null models. We then tested our hypotheses with the TYPE = TWOLEVEL RANDOM function in an integrated path-analytic approach to test multilevel moderated mediation hypotheses (Preacher et al., 2010). We relied on the 95%-confidence interval for testing conditional indirect effects. The conditional indirect effect can be interpreted as significant if the 95%-confidence interval does not include zero and not significant when it includes zero.

**Figure 2:**
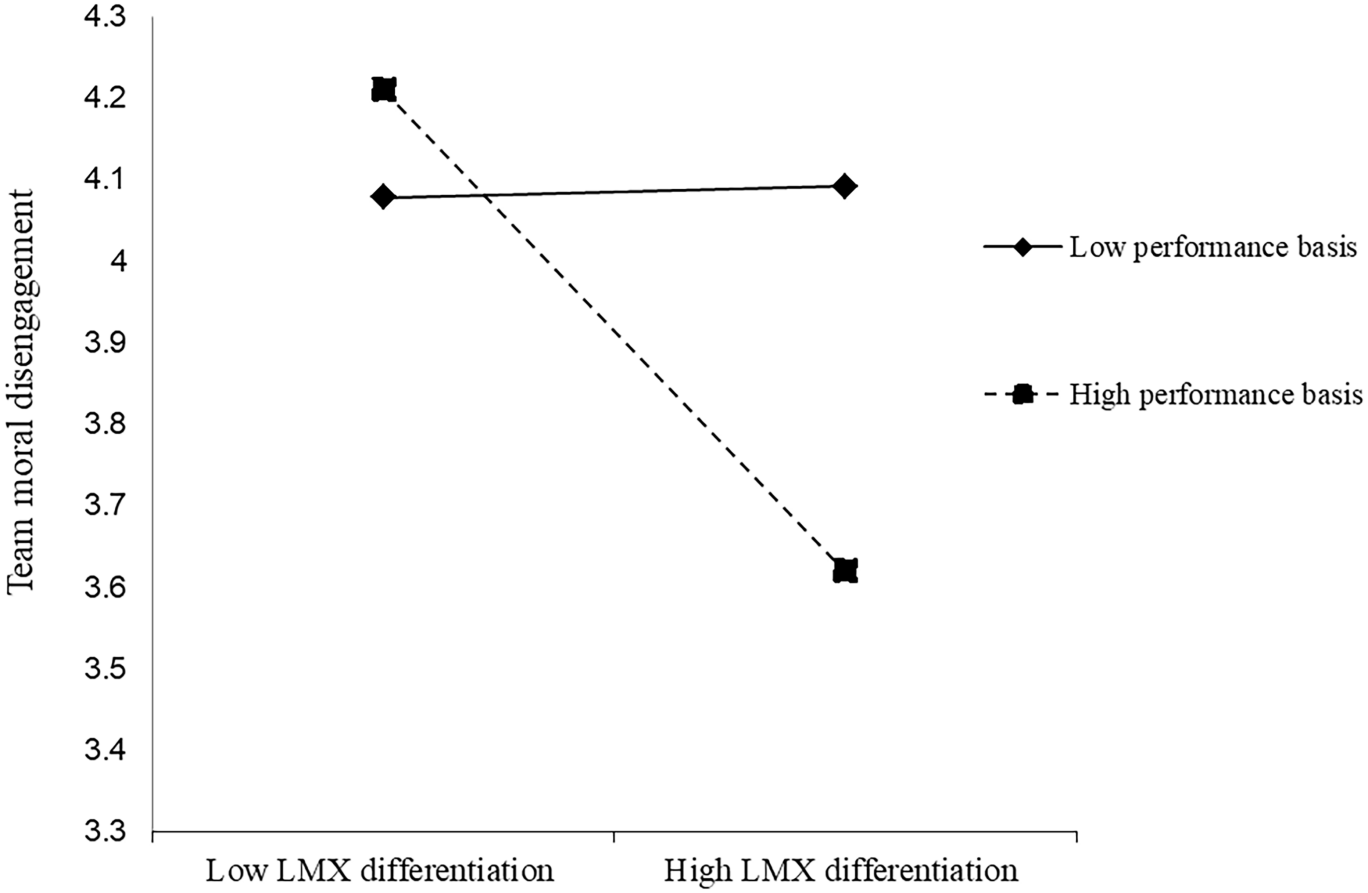
Interaction between LMX differentiation and performance basic.

**Figure 3:**
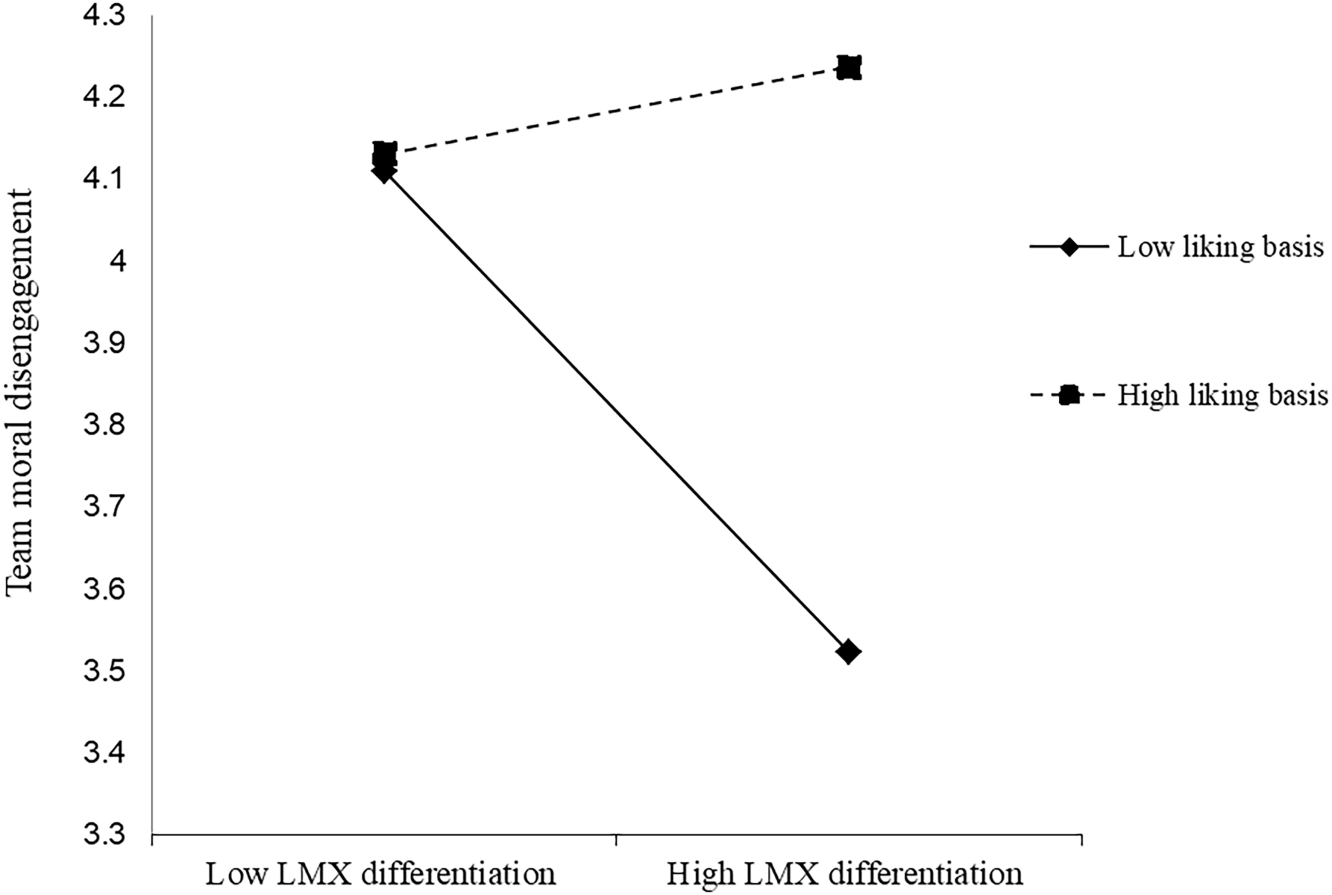
Interaction between LMX differentiation and liking basic.

Our null model’s data support the significance of between-group variation in our dependent variables, showing that COCD had 45% and CODD had 27% between-group variance. These findings indicated that the use of multilevel analysis for the current study is justified. Table 2 presents the multilevel path analysis results of our proposed hypotheses. It is noticed from the data of Table 2 that LMX differentiation does not have a significant relationship with the mediating variable or the dependent variables. Specifically, our results show that LMX differentiation was not significantly related to team moral disengagement (*β* = 0.01, n.s.), COCD (*β* = −0.48, n.s.), and CODD (*β* = −1.37, n.s.), supporting the idea of the contingent nature of this relationship. Hypothesis 1 suggested that performance basis moderated the relationship between LMX differentiation and team moral disengagement. The results supported Hypothesis 1 by showing that the interactions between performance basis and LMX differentiation were negatively related to team moral disengagement (Model 2: *β* = −1.46, *p* < 0.01). By following recommended procedures from Aiken and West (1994) (Figure 2) and the slope tests show that when performance basis was high (1 SD higher than the mean), LMX differentiation was negatively related to team’ moral disengagement (simple slope = −2.69, *p* < 0.05); meanwhile, when performance basis was low (1 SD lower than the mean), the relationship between LMX differentiation and team moral disengagement was positive and not significant (simple slope = 0.07, n.s.). Therefore, Hypothesis 1 was supported. Hypothesis 2 proposed that the personal liking basis moderated the relationship between LMX differentiation and team moral disengagement. Table 2 shows that the interactions between personal liking basis and LMX differentiation positively relate to team moral disengagement (Model 3: *β* = 1.20, *p* < 0.05). Figure 3 and the slope tests show that when personal liking basis was low (1 SD lower than the mean), LMX differentiation was negatively related to team’ moral disengagement (simple slope = −2.66, *p* < 0.05); meanwhile, when personal liking basis was high (1 SD higher than the mean), the relationship between LMX differentiation and team moral disengagement was positive and not significant (simple slope = 0.47, n.s.). Therefore, Hypothesis 2 was supported.

**Table 2:** Results of multilevel path model.

Independent variables	Collective moral disengagement			COCD	CODD
	Model1	Model2	Model3	Model4	Model5
Individual level					
Gender				0.00	0.00
Age				−0.01	−0.30
Education				−0.00	−0.02
RLMX				0.02	0.14
Team level					
Team size	−0.03	0.06	0.06	0.00	0.09
Leader gender	−0.01	−0.00	−0.00	0.01	−0.01
Leader age	−0.16	−0.06	−0.05	0.13	−0.23
Leader education	−0.05	−0.05	−0.05	0.06	−0.10
LMX mean	−0.69^**^	−0.62^**^	−0.55^*^	−0.21	−0.34
LMX differentiation	0.01	−1.31	−1.09	−0.48	−1.37
Team moral disengagement				0.23^*^	0.58^**^
Performance basis (PB)		−0.09			
Personal liking basis (LB)			0.14		
PB * LMX differentiation		−1.46^**^			
LB * LMX differentiation			1.20^*^		

* *p* < 0.05;

** *p* < 0.01.

Individual level *N* = 289, team level *N* = 76. Unstandardized regression coefficients are reported. RLMX, relative leader-member exchange; LMX, leader–member exchange; COCD, customer-oriented constructive deviance; OCDD, customer-oriented destructive deviance.

Team moral disengagement was expected to predict member outcomes. As shown in Model 4 and 5 of Table 2, team moral disengagement had a significantly positive cross-level relationship with COCD (*β* = 0.23, *p* < 0.05) and CODD (*β* = 0.58, *p* < 0.01). These findings provide support for Hypothesis 3 and 4.

Hypotheses 5a and 5b predicted that performance basis moderates the indirect effect of LMX differentiation on team member’s outcomes *via* team moral disengagement. Regarding Hypothesis 5a, the indirect effect of LMX differentiation on CODD *via* team moral disengagement was significant (indirect effect = −1.55, 95% CI = [−2.85, −0.26]; confidence interval does not contain 0) when performance basis was high, but non-significant (indirect effect = 0.05, 95% CI = [−0.66, 0.75]; confidence interval does contain 0) when performance basis was low. These findings provide support to Hypothesis 5a. However, Hypothesis 5b was not supported due to the non-significant indirect effect of the interaction of LMX differentiation × performance basis on COCD. Therefore, Hypothesis 5 was partially supported.

Hypotheses 6a and 6b suggested that personal liking basis moderates the indirect effect of LMX differentiation on team member’s outcomes *via* team moral disengagement. Regarding Hypothesis 6a, the indirect effect of LMX differentiation on CODD *via* team moral disengagement was significant (indirect effect = −1.54, 95% CI = [−2.89, −0.19]; confidence interval does not contain 0) when liking basis was low, but non-significant (indirect effect = 0.28, 95% CI = [−0.56, 1.11]; confidence interval does contain 0) when liking basis was high. These findings provide support to Hypothesis 6a. However, Hypothesis 6b was not supported due to the non-significant indirect effect of the interaction of LMX differentiation × personal liking basis on COCD *via* team moral disengagement. Therefore, Hypothesis 6 was partially supported. Detailed results of the conditional indirect effects are available on request from the first author.

## Discussion and conclusion

### Theoretical implications

Our research makes several noteworthy contributions to the literature on LMX differentiation and moral disengagement.

#### Contributions to the LMX differentiation literature

First, we contribute to the growing literature on LMX differentiation by revealing the cognitive pathway through which LMX differentiation may be associated with individual outcomes. As mentioned earlier, researchers have recently devoted significant efforts to understand the nature of the relationship between LMX differentiation and various work outcomes (e.g., Hooper and Martin, 2008; Henderson et al., 2009; Erdogan and Bauer, 2010; Le Blanc and González-Romá, 2012). Although these results illuminate many previously unclear aspects of the LMX literature, they are marked by fragmentation and inconsistency (Anand et al., 2011; Matta and Van Dyne, 2020). Some studies attribute these inconsistencies to issues related to the theoretical perspectives adopted by previous studies. Specifically, previous studies limited their interpretations of the consequences of LMX differentiation by employing specific perspectives, such as the theories of justice, social comparison, and social identity, and may not have considered alternative theoretical lenses that could illuminate hidden aspects in this context (Harris et al., 2014; Martin et al., 2018). Our paper proposed that one theoretical perspective that can provide new insights regarding the potential consequences of LMX differentiation is the moral disengagement perspective (Bandura, 1986, 1999). The mediating role of moral disengagement provides new insights to explaining the relationship between LMX differentiation and employees’ behaviors based on individuals’ cognitive responses. Our findings indicated that disengaging from one’s own internal ethical standards helps explain how differentiated treatment by the leader, under specific circumstances, can lead to bad consequences in the workplace. Therefore, the mediating role of team moral disengagement supports the view of some researchers (Martin et al., 2018; Dong et al., 2020; Matta and Van Dyne, 2020) that the relationship between LMX differentiation and outcomes cannot be limited to specific explanations related, for example, to justice or social comparison, there are other mechanisms that future studies can explore. Furthermore, our results provide the first empirical step that reveals whether and when LMX differentiation affects team moral disengagement.

Second, our findings show that LMX differentiation has no direct effect on team moral disengagement. Instead, our results reveal that LMX differentiation is negatively related to team moral disengagement when the performance basis is high or when the personal liking basis is low. These results support the idea that part of the inconsistent results of prior studies may be from overlooking boundary conditions that can make outcomes of differentiation positive or negative (Martin et al., 2018; Matta and Van Dyne, 2020). By examining the moderating role of two bases of LMX differentiation—performance and personal liking—we further extend the current research that sees that judging the fairness or unfairness of the leader’s treatment is what determines whether LMX differentiation’s outcomes are good or bad (Chen et al., 2018; Matta and Van Dyne, 2020; Han et al., 2021). Past studies showed that when leaders differentiated LMX based on high performance-related factors, the relationship between LMX differentiation and group cooperation (Han et al., 2021) and procedural justice (Chen et al., 2018) were positive. However, when differentiation was based to a greater extent on personal liking-related factors or based less on performance-related factors, these relationships were negative. Accordingly, our hypothesized model expands the scope of this research by dealing with alternative mechanisms, such as moral disengagement.

Third, the results of our study showed that LMX differentiation has cross-level effects on members’ outcomes (COCD and CODD). Although many researchers have emphasized that LMX differentiation is a complex, multifaceted phenomenon whose essence can only be understood when examining it from multiple levels (e.g., Henderson et al., 2008; Anand et al., 2011), a large number of previous research has been confined to the group level (Yammarino et al., 2005; Anand et al., 2011; Harris et al., 2014), with the notable exception of the use of multilevel frameworks by some studies (Estel et al., 2019; Choi et al., 2020; Eichenseer et al., 2020). Our paper helps create more comprehensive predictions about LMX differentiation outcomes by being one of a few studies empirically testing from a multilevel perspective.

#### Contributions to the moral disengagement literature

We also contribute to the literature on moral disengagement in several ways. First, the vast majority of research on the relationship between leadership behavior and moral disengagement has focused on behaviors that reduce moral disengagement, such as ethical leadership (e.g., Palmer, 2013; Huang and Yan, 2014; Moore et al., 2019), leaving leadership behaviors that can increase it less well-known (Newman et al., 2020). Our findings suggest that research on moral disengagement should move beyond models that only include leadership behaviors that lead to or terminate moral disengagement to focus on the boundary conditions that govern this relationship. Indeed, our work indicates that when a leader’s behavior differentiates team members based on performance-related inputs, their moral disengagement is low, and when a leader’s behavior differentiates team members based on personal liking-related inputs, their moral disengagement is high. This approach can give a deeper insight into the influence of leaders on members’ moral disengagement. Second, extant studies are mainly based on an individual-level conceptualization of moral disengagement. Group-level research on moral disengagement is rare (Ogunfowora et al., 2021). Thus, in our study, we answer scholarly calls (e.g., Newman et al., 2020; Ogunfowora et al., 2021) to examine the antecedents and consequences of collective moral disengagement.

Third, our contribution is not only limited to the antecedents of moral disengagement but also includes the consequences. Most studies of moral disengagement have looked at a narrow range of outcomes, specifically outcomes limited to how morally disengaged organizational members misbehave toward those with whom they have lateral relationships within the organization, such as employees, subordinates, and superiors (e.g., Astrove et al., 2015; Fida et al., 2015; Zheng et al., 2019). However, Johnson and Buckley (2015) indicated that scholars need to explore other outcomes to move forward with moral disengagement research, such as how morally disengaged organizational members act up toward those with whom they have relationships outside the organization’s boundaries, such as customers. Our research responds to Johnson and Buckley's (2015) call to explore the relationship between moral disengagement and employees’ behaviors toward customers. To our knowledge, our research is among the first to investigate how team moral disengagement can lead employees to adopt deviant behavior toward customers.

### Practical implications

Leaders are encouraged to realize that differentiation in the treatment between team members in and of itself is not bad but, in a given context, may be an important tool for making efficient use of their resources and time, as well as a means to achieve a better fit between employees’ ability and their work requirements (Anand et al., 2011; Martin et al., 2018). Nevertheless, this bright side may only be achieved when they use the right factors to base the quality of their relationship with team members. More so, leaders should keep in mind that members are susceptible to differentiation and may ask themselves from time to time what is the reasoning or the basis on which the leader brings a particular group of members closer while excluding others. Leaders need to know that team members will respond favorably to this differentiation when they perceive that it is based on performance-related factors, such as ability and skill. Conversely, when differentiation is based on criteria linked to personal liking, they will respond unfavorably. Therefore, we strongly recommend that leaders adopt performance-related factors and stay away from personal matters in building the quality of their relationship with members. Since LMX is subjective to a member’s assessment of the quality of the relationship with their leader, leaders should consider in this context effective mechanisms for making the differences in LMX observable to team members. This mean that leaders who try to establish a differentiation based on performance-related criteria should make behavioral representations that express their priorities in establishing these criteria. For example, they should be clear and consistent in their behavior over time and situations in having positive dealing with those members with high performance and experience and avoiding any behavior that could confuse the accuracy of this perception by members.

The findings indicate that organizations need to follow vigilantly the basis on which leaders distribute their resources and time toward team members and urge them to make allocations based on factors related to performance and efficiency, rather than personal liking. This is because differentiation based on personal liking can incur many costs for the organization, such as the loss of customers or the loss of the desired employee behavior. The immediate fear that the organization and its leaders should be aware of is that when leaders’ differentiation is based on performance-irrelevant inputs (for example, personal liking, similarity, or compatibility), members will break free from their moral standards and act unethically easily without feeling any psychological pain or social pressure.

### Limitations and future research

As with all research, our paper includes a number of limitations that we hope to address in future studies. First, collecting our variables’ data at the same point in time prohibits us from making any causal inferences. The cross-sectional nature of our data is a worrying factor that should be addressed in future studies by adopting a longitudinal design. Second, our sample consisted only of frontline employees working in the banking sector. Although we assume that the proposed relationships between study variables hold across different cultures and sectors, the results of our research should be replicated in other contexts and samples to confirm external validity. Third, Dulebohn et al. (2012) have indicated that three factors can be a basis for LMX differentiation: the characteristics of the follower, the leader, and their interaction. Since we focused on performance and personal liking as the two primary sources of LMX differentiation, undiscovered bases for LMX differentiation such as personality and different demographic variables could be fruitful content for future studies. Fourth, although our study has justified the reasons for relying on standard deviation as a measure of LMX differentiation, this measure may oversimplify the phenomenon because it captures only the degree of differentiation, failing to consider the effect of the configurations of LMX within the teams. Recent research suggests that both high and low differentiation can lead to negative outcomes (like team moral disengagement), depending on how LMX relationships are distributed in the groups (Seo et al., 2018; Buengeler et al., 2021). We invite future studies to take this limitation into account. Fifth, fairness (or justice) is an important mechanism in our arguments for the effect of LMX differentiation on team moral disengagement. However, it is not measured and integrated in our model. While we think the presence of a potential unmeasured mechanism in our model is acceptable, we recommend future studies to address this limitation by exploring the mechanisms in more details.

## Data availability statement

The raw data supporting the conclusions of this article will be made available by the authors, without undue reservation.

## Ethics statement

Ethical review and approval was not required for the study on human participants in accordance with the local legislation and institutional requirements. Written informed consent for participation was not required for this study in accordance with the national legislation and the institutional requirements.

## Author contributions

AA-A presented the idea of the paper and contributed to writing the introduction, hypotheses, and data analysis. EA collected data and assisted in writing the study’s hypotheses. AB, MD, and HS contributed to synthesizing and writing the theoretical evidence that supports the study’s hypotheses, and its theoretical and practical implications, as well as contributing to various details of the paper. All authors contributed to the article and approved the submitted version.

## Conflict of interest

The authors declare that the research was conducted in the absence of any commercial or financial relationships that could be construed as a potential conflict of interest.

## Publisher’s note

All claims expressed in this article are solely those of the authors and do not necessarily represent those of their affiliated organizations, or those of the publisher, the editors and the reviewers. Any product that may be evaluated in this article, or claim that may be made by its manufacturer, is not guaranteed or endorsed by the publisher.
